# The landscape of mitophagy in sepsis reveals PHB1 as an NLRP3 inflammasome inhibitor

**DOI:** 10.3389/fimmu.2023.1188482

**Published:** 2023-06-08

**Authors:** Shipeng Chen, Jinqi Ma, Ping Yin, Fang Liang

**Affiliations:** ^1^ Department of Hematology and Critical Care Medicine, The Third Xiangya Hospital, Central South University, Changsha, Hunan, China; ^2^ Cancer Research Institute, Central South University, Changsha, Hunan, China; ^3^ Department of Blood Transfusion, The Third Xiangya Hospital, Central South University, Changsha, Hunan, China

**Keywords:** mitophagy, PHB1, NLRP3 inflammasome, sepsis, immunity

## Abstract

Mitophagy is a selective autophagy targeting damaged and potential cytotoxic mitochondria, which can effectively prevent excessive cytotoxic production from damaged mitochondria and alleviate the inflammatory response. However, the potential role of mitophagy in sepsis remains poorly explored. Here, we studied the role of mitophagy in sepsis and its immune heterogeneity. By performing mitophagy-related typing on 348 sepsis samples, three clusters (A, B, and C) were obtained. Cluster A had the highest degree of mitophagy accompanied by lowest disease severity, while cluster C had the lowest degree of mitophagy with the highest disease severity. The three clusters had unique immune characteristics. We further revealed that the expression of PHB1 in these three clusters was significantly different and negatively correlated with the severity of sepsis, suggesting that PHB1 was involved in the development of sepsis. It has been reported that impaired mitophagy leads to the over-activation of inflammasomes, which promotes sepsis development. Further analysis showed that the expressions of NLRP3 inflammasomes core genes in cluster C were significantly up-regulated and negatively correlated with PHB1. Next, we verified whether PHB1 downregulation caused the activation of inflammasomes and found that the PHB1 knockdown increased the levels of mtDNA in the cytoplasm and enhanced the activation of NLRP3 inflammasomes. In addition, mitophagy inhibitor treatment abolished PHB1 knockdown-mediated activation of NLRP3 inflammasomes, suggesting that PHB1 inhibited the activation of inflammasomes through mitophagy. In conclusion, this study reveals that a high degree of mitophagy may predict a good outcome of sepsis, and PHB1 is a key NLRP3 inflammasome regulator *via* mitophagy in inflammatory diseases such as sepsis.

## 1 Introduction

Autophagy is a self-protection mechanism that can quickly clear away the redundant and damaged organelles in cells to avoid apoptosis and necrosis, thus maintaining the stability of the intracellular environment (1, 2). Autophagy is usually divided into non-selective (a starvation reaction) and selective autophagy (maintaining cell homeostasis) according to whether it is selective to substrates. The dysfunction of selective autophagy has been associated with the occurrence of a series of diseases in human (3). Mitochondrial autophagy (mitophagy) is an important selective autophagy that can undergo depolarization and damage under the stimulation of reactive oxygen species (ROS), nutrient deficiency, hypoxia, and inflammatory factors. These damaged, aged and dysfunctional mitochondria were selectively transported by the autophagy system to lysosome for degradation (4). Mitophagy-mediated elimination of damaged mitochondria plays an important role in the survival and development of organisms, such as embryonic development, inflammation, cell differentiation and apoptosis (5). It has been reported that abnormal mitophagy is associated with various diseases, such as neurodegenerative disease (6), cardiovascular disease (7), liver disease (8) and cancer (9). Sepsis is a syndrome of physiological, pathological and biochemical abnormalities caused by infection and the excessive activation of host response (10), and has the characteristics of high incidence, extremely heterogeneous, rapid development and easy to cause multiple organ failure. Prompt diagnosis and appropriate treatment are extremely important for patients with sepsis (11). However, due to a limited understanding of the pathogenesis of sepsis and the lack of effective treatment, sepsis is still the leading cause of death in intensive care unit (ICU). A recent meta-analysis result showed that the mortality rate in ICU was as high as 42% (12). In general, sepsis can cause inflammatory hyperactivation, immune dysfunction and coagulation disorders, leading to tissue or organ damage (13). Due to the complexity of host inflammatory reaction and the differences of clinical research (14) it is still necessary to develop more precise therapeutic strategies. It was reported that improving mitochondrial damage can reduce the early and long-term mortality of patients with sepsis (15). Mitophagy plays a vital role in controlling mitochondrial integrity and maintaining mitochondrial dynamic balance (16). In addition, mitophagy disorder is associated with overactivation of inflammasomes during progression of sepsis (17), which was induced by ROS production (18), mitochondrial dysfunction (19), and translocation of mitochondrial DNA into the cytoplasm (20). Therefore, mitophagy-mediated inflammasome activation has a potential role in sepsis. However, relevant reports are still limited, and the mechanism has not yet been clarified. In this study, we aim to explore the role of mitophagy in sepsis, and further verify it with experiments.

## 2 Materials and methods

### 2.1 Data sets acquisition

R 4.0.3 was used for data mining and analysis. The sepsis-related dataset GSE185263 (21) was obtained from Gene Expression Omnibus (GEO, https://www.ncbi.nlm.nih.gov/geo/) by R package “GEOquery”. The whole blood RNA-seq data and clinical data of 392 samples were collected in this cohort, including 348 patients from emergency room (ER) or intensive care unit (ICU) and 44 normal samples. Annotation conversion was performed on the gene probes of the raw data, and the count data was further converted into tpm data for subsequent analysis.

### 2.2 Construction of mitophagy-related signature set

First, in 348 sepsis samples, gene module with a high correlation pattern (excluding grey modules that are not clustered) were identified by using WGCNA (Weighted Gene Co-expression Network Analysis) (22). According to the mitophagy-related pathways score, the WGCNA module named MEgreenyellow which might be most related to mitophagy was obtained. The 926 high-correlation top genes in MEgreenyellow module were further obtained (Gene Significance for Mitophagy **≥** 0.5 & Module Membership in Greenyellow module **≥** 0.8). In addition, 230 mitophagy-related genes (Relevance score **≥** 1.5) were identified through retrieval “mitophage” from GeneCards (https://www.genecards.org/). Finally, 22 mitophagy genes of top genes in the module were as the signature set related to mitophagy in sepsis.

### 2.3 Quantization function analysis and enrichment analysis

single sample Gene Set Enrichment Analysis (ssGSEA) was used to quantify the score of the mitophagy (mitochondrial autophagy) signature set for each sample to assess the extent of mitophagy enrichment for different samples. MA score was introduced to quantify mitophagy signature set of each sample by ssGSEA. The enrichment scores of related pathways between different samples were compared by Gene Set Variation Analysis (GSVA) (23). Gene Set Enrichment Analysis (GSEA) (24) was used to analyze the enrichment of biological functions between different clusters, and limma package was used to calculate the fold change of expression difference of genes between groups. Background gene sets (C2: curated gene sets, C5: ontology gene sets) were provided by Molecular Signals Database (MSIGDB) (https://www.gsea-msigdb.org/gsea/index.jsp). In addition, Metascape (http://metascape.org/) (25) was also used for biological process-related enrichment analysis, p value < 0.01, minimum count of 3, and enrichment factor of term > 1.5 were collected, and the entry nodes were clustered according to the member similarity.

### 2.4 Mitophagy clustering (Consensus Clustering)

Consensus clustering, an unsupervised clustering method, classifies samples into several clusters based on omics data set, and can compare and analyze different clusters. The R package “ConsensusClusterPlus” (26) was used for clustering of sepsis samples. Through the combination of different algorithms, the clustering algorithm (k-means) and the distance algorithm (euclidean) were finally determined. When k=3, the samples could be stably clustered into three cluster patterns. Biomarker comparisons between clusters are shown by the R package “ComplexHeatmap” (27).

### 2.5 Modularization analysis of gene interaction network

STRING (https://cn.string-db.org/) (28) database was used to analyze the interaction network of mitophagy-related genes, Metascape (http://metascape.org/) database was used for a further modular analysis of gene interaction network, and Cytoscape (https://cytoscape.org/) (29) was used for further optimization of gene interaction network.

### 2.6 Cluster immune score

The immune scores of different samples were calculated using different immune algorithms of R package “IOBR” (30) to quantify and evaluate the abundance of different types of immune cells in peripheral blood between different clusters. We integrated a total of six immune algorithms (CIBERSORT, EPIC, MCP_counter, Quantiseq, TIMER, xCell) to classify the cell types into four different sources (lymphoid, myeloid, stem cells, and stromal cells). Among them, MCP-counter and xCell are based on marker genes expression, while CIBERSORT, EPIC, Quantiseq and TIMER are based on deconvolution algorithm to quantify the abundance of immune cells among different clusters. Then, we portrayed the immune characteristics of different clusters with the four types of cells. In addition, only cell types whose abundance of immune cells with statistical significance among different subtypes are shown in our results (p < 0.05).

### 2.7 RNA interference assay

To obtain mouse primary peritoneal macrophages, mice were injected intraperitoneally with 3% thioglycolate. Three days later, peritoneal exudate cells were harvested and incubated. Two hours later, nonadherent cells were removed and the adherent monolayer cells were used as peritoneal macrophages. For silencing of PHB1, mouse primary peritoneal macrophages were seeded in 12-well (5 × 10^5^ cells per well), then transfected with siRNA using Lipofectamine RNAiMAX (Thermo Fisher Scientific) according to the manufacturer’s instructions. The siRNA sequences for mouse PHB1 (siRNA1: 5’-CGUGGUGAACUCUGCUUUGUATT-3’; siRNA2: 5’-CGUCAAUAUCACACUGCGAAUTT-3’; siRNA3: 5’-GAGCCAGAUUU GUGGUGGAAATT-3’) and the negative control (UUCUCCGAACGUG UCACGUTT) were chemically synthesized by Sangon Biotech Co., Shanghai, China.

### 2.8 Immuno-blot

Cell lysates were prepared using RIPA lysis buffer and protein concentration was determined by the BCA method (Thermo Fisher Scientific). Samples were separated by 12% SDS-PAGE and transferred onto PVDF membranes (Millipore). Antibody to PHB1 (Abcam) was used at 1:1000 dilution. Blots were normalized to GAPDH expression.

### 2.9 Cell stimulation

For NLRP3 inflammasome activation, mouse primary peritoneal macrophages were primed with LPS (100 ng/mL) for 3 h followed by stimulation with 5 mM ATP (30min) or 10 μM Nigericin (1h). For mitophagy inhibition, cells were pre-treated with 5 mM 3-MA (1h). Supernatants were collected for ELISA detection.

### 2.10 Measurement of IL-1b and IL-8

The level of IL-1b and IL-18 in cell culture supernatant were analyzed using IL-1b ELISA kit (eBioscience) and IL-18 (Abcam) according to the manufacturer’s instructions.

### 2.11 Isolation and quantification of cytosolic mitochondrial DNA

mtDNA was isolated from the cytosolic fractions using a DNeasy Blood & Tissue Kit (Qiagen). The relative mtDNA levels were determined by qPCR with the primers of NADH dehydrogenase 6 (ND6) gene. Forward primer (5’-TTAGCATTAAAGCCT TCACC-3’) and reverse primer (5’-TAACAATCACCCAAACAACC-3’) of ND6 were used. Quantitative PCR was performed using SYBR Green (Vazyme Biotech) on a LightCycler 480 (Roche Diagnostics).

### 2.12 Screening of drugs binding to PHB1

First, the ubiquitination site was identified by using PhosphoSitePlus (https://www.phosphosite.org/homeAction) (31) to predict the post-translational modification of PHB1 protein. Next, structural files for the 9,468 listed drugs were obtained through the DrugBank database (https://go.drugbank.com/) (32). The PHB1 structure file is obtained from the Alphafold protein structure database (https://alphafold.ebi.ac.uk/) (33). We further docked the compounds to the target protein using Autodock Qvina2 (https://vina.scrips.edu/) (34) and screened candidate compounds by assessing protein-ligand affinity. The interactions between PHB1 and its ligands were further analyzed by using PLIP (https://plip-tool.biotec.tu-dresden.de/plip-web/plip/index) (35).

### 2.13 Quantification and statistical analysis

The Wilcoxon test was used to compare non-normally distributed variables between unpaired groups. The Kruskal-Wallis test was used to compare non-normally distributed variables between groups. Pearson method was used for correlation analysis. For all experiments, the number of independent experiments (n) were described in the legend. Two-tailed unpaired Student’s t test was used to compare the differences between two groups. Two-way ANOVA tests were used followed by *post hoc* Bonferroni test for multiple comparisons. Statistical analysis was performed using R 4.0.3 and GraphPad Prism software 8.0. A p value < 0.05 was considered statistically significant for all analyses. *P<0.05, **P<0.01, ***P<0.001, ****P<0.0001.

## 3 Results

### 3.1 Construction of mitophagy signature set in sepsis cohort

**Figure 1:**
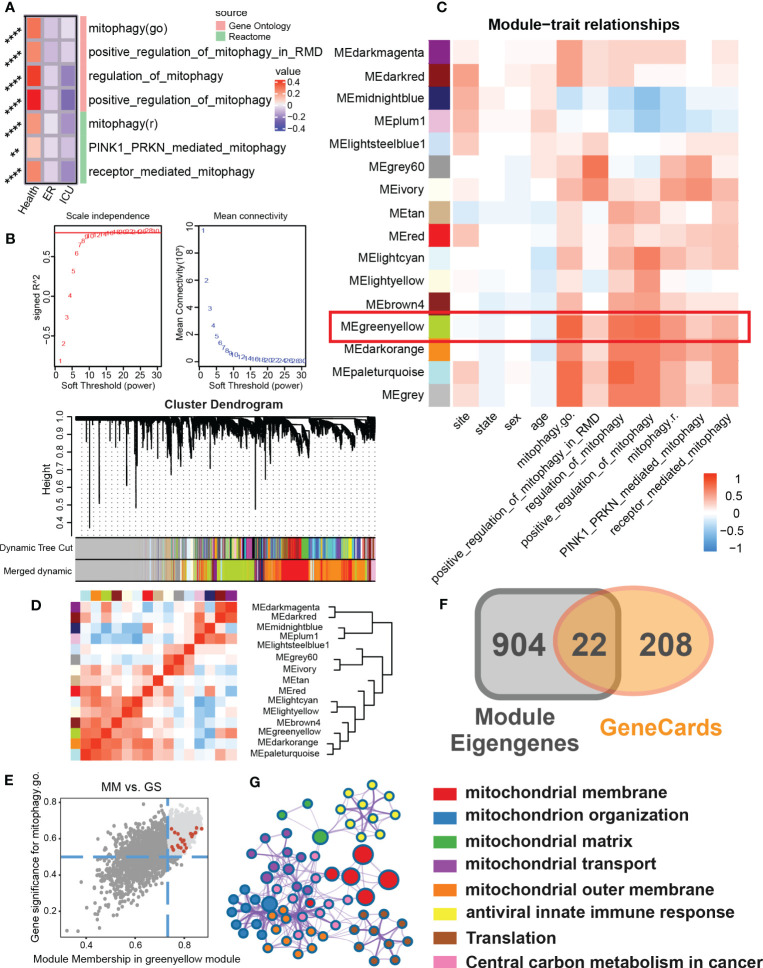
To identify the mitophagy signature set in a sepsis cohort. **(A)** The heat map showed GSVA scores for mitophagy-related items of Gene Ontology and Reactome for different subgroups of clinical features (Health, ER, ICU) in the sepsis cohort. **(B)** Scale independence and mean connectivity related to WGCNA soft threshold screening. Cluster denogram for module identification. **(C)** Module-trait relationships. The heat map showed the correlation between the different modules and the clinical features as well as the mitophagy score. **(D)** Eigengene dendrogram and Eigengene adjacency heatmap showed similarities between different modules. **(E)** Scatter plot of module membership(MM) and gene significance(GS) of eigengene for Module MEgreenyellow. And screening top genes with high GS and high MM. **(F)** The Venn map that identified the mitophagy signature set in sepsis. The module eigengenes with genes with high GS and high MM shared 22 genes with the mitophage-related genes in GeneCards. **(G)** The biological process enrichment network of the mitophagy signature set. ** P<0.01, **** P<0.0001.

To test whether the mitophagy activity of sepsis patients is changed, we first evaluated the enrichment of mitophagy-related pathways in emergency room (ER) or intensive care unit (ICU) samples (**Figure 1A**). We found that compared with the healthy population, the mitophagy activity in sepsis patients was generally down-regulated, specifically in the positive regulation of mitophagy in ICU patients, indicating that mitophagy may play an important role in sepsis patients. To further explore the role of mitophagy in sepsis, we clustered sepsis samples into 15 non-gray modules by WGCNA analysis (**Figures 1B–D**). After analyzing the correlation between different modules, the clinical characteristics of samples and the score of mitophagy pathway, we found that MEgreenyellow module had a high correlation with multiple mitophagy pathways (**Figure 1C**). After further focusing on the MEgreenyellow module, 926 genes highly associated with mitophagy pathways (gene significance > 0.5 and Module membership > 0.8) were selected to intersect with mitophagy-related genes, and finally obtained 22 mitophagy-related hub genes in sepsis patients (**Figures 1E, F**). These 22 hub genes were mainly enriched in a variety of mitochondrial biogenesis-related modules (mitochondrial membrane, mitochondrial organization, mitochondrial matrix, mitochondrial transport, central carbon metabolism, etc.) (**Figure 1G**).

### 3.2 Three mitophagy clusters were identified in a sepsis cohort based on the signature set of mitophagy

**Figure 2:**
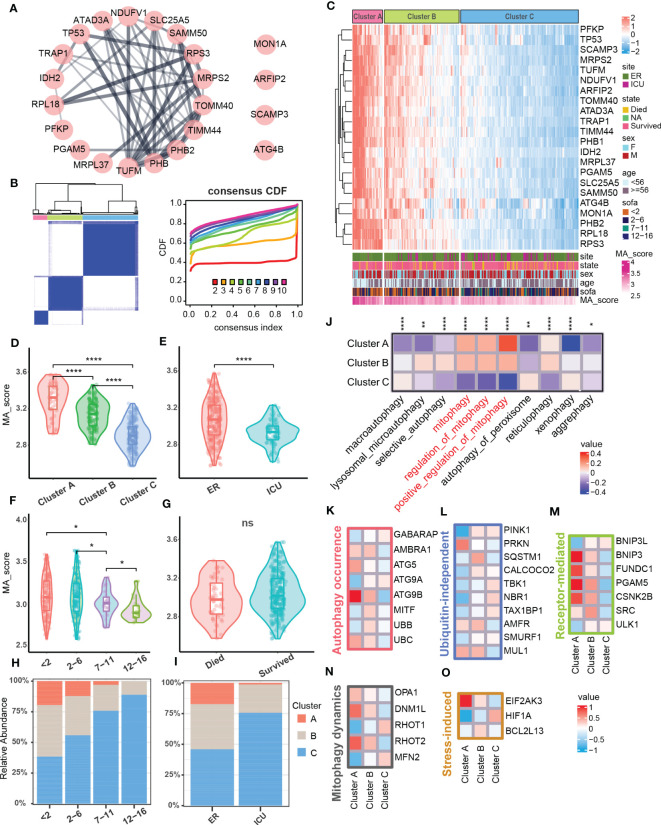
Mitophagy clustering in sepsis cohort. **(A)** Protein-protein interaction network related to mitophagy signature set. **(B)** Consensus matrix heatmap. Consensus cumulative distribution diagram. **(C)** Heatmap showed gene expression level of mitophagy signature set and mitophagy clustering in the sepsis cohort (*N* = 348). The bar graph below the heat map showed the clinical features and the distribution of the MA score. **(D–G)** Violins showed MA score between mitophagy clusters and subgroups of clinical features (ER/ICU, SOFA, State). **(H)** The bar graph showed the distribution of mitophagy clusters across subgroups of SOFA scores. **(I)** The bar graph showed the distribution of mitophagy clusters in the ER and ICU subgroups. **(J)** Heatmap showed GSVA scores of autophagy-related pathways between different clusters. **(K–O)** Heatmap respectively showed the expression of marker related to different molecular pathways in mitophagy among different clusters, and the expressions of all screened marker in different clusters were statistically significant (p<0.05). ns≥0.05, * P<0.05 , ** P<0.01, **** P<0.0001.

Since the degree of mitophagy in sepsis patients was generally down-regulated compared with the healthy population, we next tested the difference in the expression of mitophagy-related genes among sepsis patients. We further performed consistent clustering analysis on the obtained 22 key mitophagy-related genes in sepsis samples (**Figure 2A**), and found that mitophagy genes in different sepsis samples could be stably clustered into 3 clusters (**Figure 2B**). We took the mitophagy-related hub genes enrichment scores as a standard MA score for evaluating the mitophagy activity in different samples. Obviously, hub genes for mitophagy was highly abundant in Cluster A, while MA score in Cluster C was significantly down-regulated, and Cluster B functions as a transitional cluster of mitophagy alteration (**Figures 2C, D**). Through the evaluation of different clinical characteristics, we found that the mitophagy MA score of ICU samples was lower than that of ER samples (**Figure 2E**). In addition, MA score was reduced with increased SOFA (Sequential Organ Failure Asses) score which is used to assess the severity of sepsis in clinical practice (10) (**Figure 2F**). However, the MA score was not comparable between survival and dead patients (**Figure 2G**). In addition, the higher SOFA score, the higher percentage of Cluster C. Cluster A showed an inversive relationship with Cluster C (**Figure 2H**). Similarly, Cluster A was mainly enriched in ER samples, but Cluster C was primarily enriched in ICU samples (**Figure 2I**). By assessing the enrichment of mitochondrial autophagic biological processes, we confirmed that Cluster A had the highest mitophagy activity, and Cluster C showed the lowest mitophagy activity (**Figure 2J**).

We next test the mitophagy activity induced by which molecules and cascades in Cluster A, B, and C (36). Among the autophagy-related molecules, ATG5 and ATG9B that induce the formation of autophagosomes as well as UBC in the ubiquitin family were significantly increased in Cluster A (**Figure 2K**). In ubiquitin-independent mitophagy, PRKN was significantly up-regulated in Cluster A, and AMFR and MUL1 were enriched in both Cluster A and B (**Figure 2L**). In receptor-mediated mitophagy, a variety of related molecules (BNIP3, FUNDC1, etc.) were increased in Cluster A (**Figure 2M**). In addition, the expression of mitochondrial dynamics and stress-induced related molecules in mitophagy were also changed in different clusters (**Figures 2N, O**). The above results indicated that mitophagy dysfunction might lead to different degrees of sepsis pathological conditions, and multiple molecular pathways participated in mitophagy in sepsis.

### 3.3 Immune landscape differences in mitophagy clusters

**Figure 3:**
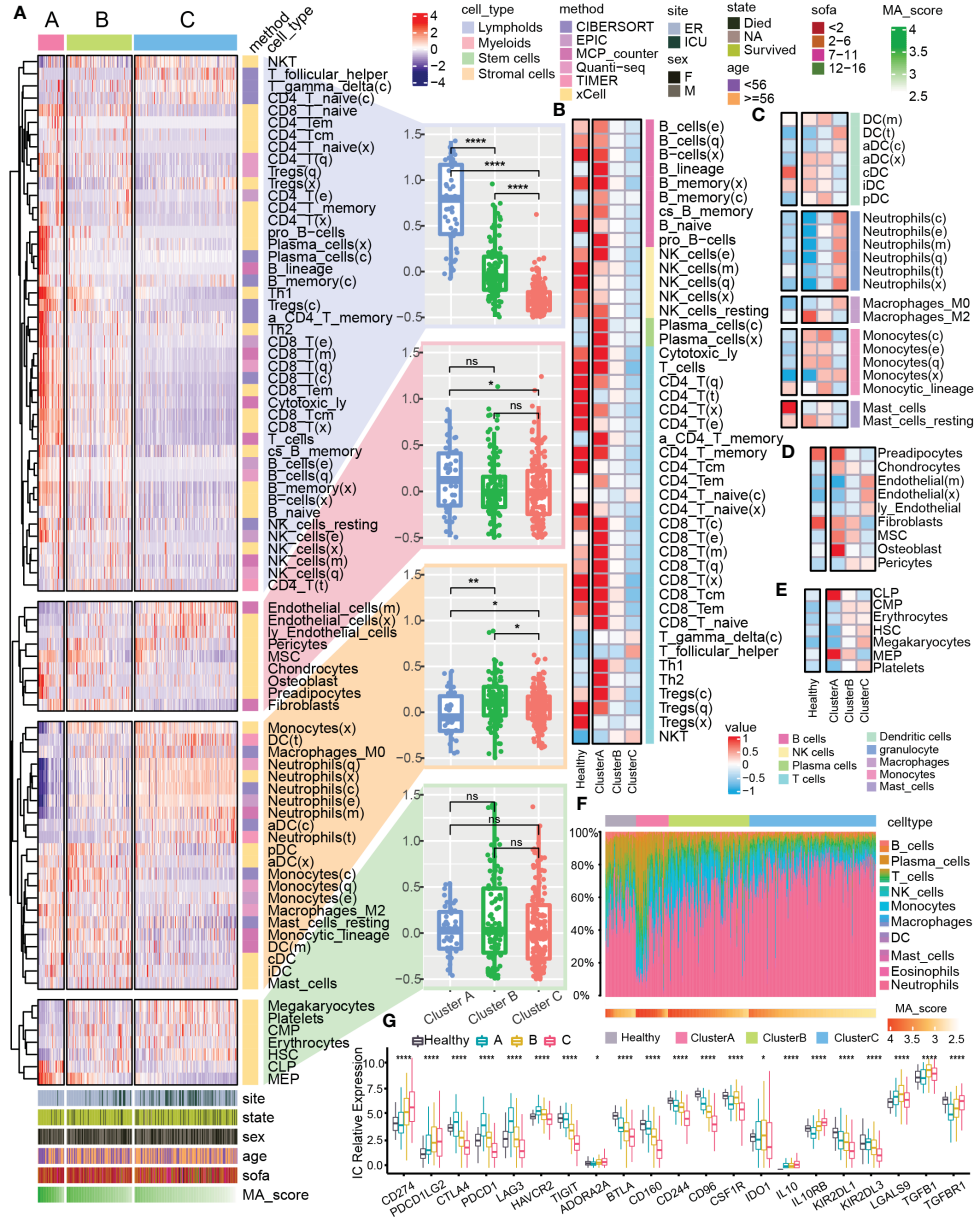
Immune landscape of mitophagy clusters. **(A)** The heat map shows the distribution of immune scores of different types of cells from the four sources in mitochondrial clusters. The bar graphs at the bottom of the heat map show the clinical features and the distribution of MA score. The bar graphs at the right of the heat map show the cell source and immune scoring algorithm. Boxplots showed the difference in the overall distribution of the scores of the cells from different sources in the three clusters, respectively. **(B–E)** Heatmap showed the differences in immune scores of the four different cell type (lymphoid, myeloid, stem, stromal) from different clusters and the control group. **(F)** Heatmap showed the proportional fraction of 22 immune cells in different clusters and control group calculated by the CIBERSORT immune algorithm. **(G)** Boxplot showed the expression of immune checkpoint related genes in different groups. ns≥0.05, * P<0.05 , ** P<0.01 , **** P<0.0001.

The pathogenesis of sepsis involves complex systemic inflammatory network effects and immune dysfunction. Therefore, we further explored the differences of immune characteristics among the clusters. We integrated multiple immune analysis algorithms and classified all the immune cells into four sources: lymphoid, myeloid, stem and stromal cells (**Figure 3A**). Lymphoid immune cells were highly enriched in Cluster A (**Figure 3A**) but was significantly reduced in Cluster C (**Figure 3B**). Different types of myeloid cells were distributed in the 3 clusters. For example, the abundance of M2 macrophages increased significantly in cluster A (**Figure 3C**), which might be related to the enhancement of anti-inflammatory response (37–39). The enrichment of stromal cells and common lymphoid progenitor cells (CLP) showed differences between clusters (**Figures 3D, E**). In the overall immune landscape (**Figure 3F**), from Cluster A to Cluster B, and then to Cluster C, the abundance of lymphoid immune cells was gradually decreased, while the abundance of myeloid immune cells was increased, which mediated the aggravation of the inflammatory response. In addition, compared with the control group, the difference in the proportion of different immune cells in Cluster B was not obviously changed. We also focused on changes in the expression of immune checkpoints in different clusters of sepsis (**Figure 3G**). Compared with the normal sample, many changes were found in expression levels of some immune checkpoint-related genes in Cluster C, for example CD274 and PDCD1LG2 were upregulated, but CTLA4 and TIGIT were downregulated, which might be related to the enhancement of inflammatory effects *in vivo* and immune dysfunction (40, 41). The dysfunction of mitophagy reflects the changes of immune characteristics to some extent.

### 3.4 As a mitophagy-related gene, PHB1 is differentially expressed among different clusters

**Figure 4:**
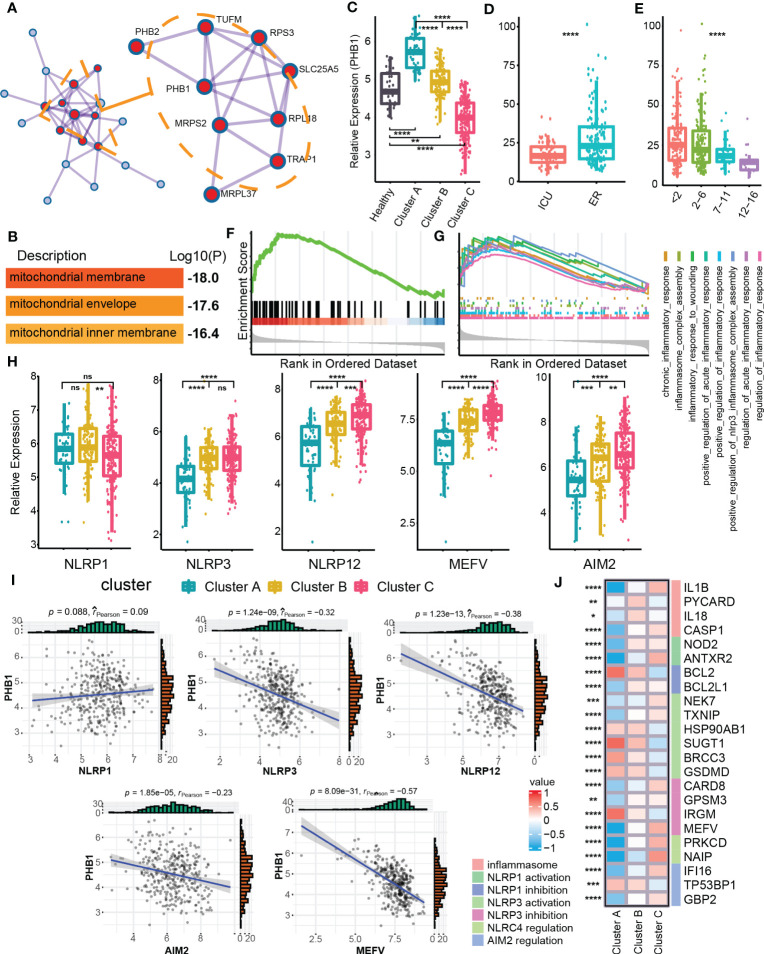
Identification of PHB1 in mitophagy Eigengene network. **(A)** Densely connected network components identified by MCODE. **(B)** The entry shows the pathway and process enrichment analysis for the network’s main MCODE component (red). **(C)** Boxplot showed the expression of PHB1 in clusters and control groups. **(D)** Boxplot showed the expression of PHB1 in different sepsis severity groups. **(E)** Boxplot showed the expression of PHB1 in different SOFA grade subgroups. **(F)** The enrichment of NOD like receptor signaling pathway in the Cluster C - Cluster **(A, G)** The enrichment of multiple inflammatory-related biological processes in the two clusters of Cluster C - Cluster **(A, H)** The boxplot showed the expressions of a variety of key genes related to inflammasomes in the clusters. **(I)** The scatter diagram showed the expression correlation of PHB1 and various inflammasome-related genes. **(J)** The heatmap showed the expression of inflammasome-related regulatory genes among the different clusters. ns≥0.05, * P<0.05 , ** P<0.01 , *** P<0.001, **** P<0.0001.

In order to identify the clusters-related core targets from mitophagy hub genes, we modularized and clustered hub genes into two modules (**Figure 4A**), where the core modules were mainly related to the biological process of mitochondrial membrane and mitochondria envelop (**Figure 4B**). Among these, we found that PHB1 was located at the core of the overall network, with the highest degree value. PHB1 is a gene related to mitophagy and plays an important role in the mitophagy (36, 42). We further examined the difference in expression of PHB1 in different clusters and clinical features. Cluster A had a higher PHB1 expression than healthy samples, and Cluster C had a lower expression than normal samples (**Figures 4C, D**). In addition, PHB1 expression was decreased with increased SOFA grades (**Figure 4E**). Cluster C showed a highly expressed classical inflammasome-related pathway, the Nod-like receptor signaling pathway compared with Cluster A (**Figure 4F**). We also focused on the expression of other inflammation-related pathways and found that the inflammatory response was highly up-regulated in Cluster C (**Figure 4G**). The changes in inflammation-related pathways were usually accompanied by changes in the expression of multiple inflammasomes (43, 44). Among the three clusters, multiple inflammasomes molecules were up-regulated in Cluster C (except for NLRP1) (**Figure 4H**). Besides, the core mitophagy molecule PHB1 also showed a negative correlation with a variety of inflammasome molecules, specifically with NLRP3, MEFV, and NLRP12 (**Figure 4I**). Since the inflammasomes have multiple molecular regulatory pathways (44), we have also explored the molecular expression of different inflammasome-related regulatory pathways in mitophagy clusters, and multiple regulatory molecules have cluster differences (**Figure 4J**). The above results further indicated that the dysfunction of mitophagy was highly correlated with inflammatory effects.

### 3.5 PHB1 inhibits the activation of NLRP3 inflammasome by regulating mitophagy

A large number of studies have shown that abnormal activation of NLRP3 inflammasome plays an important role in the pathogenesis and progression of sepsis (45). NLRP3 inflammasome is sensitive to pathogen-associated molecular patterns (PAMPs) and damage-associated molecular patterns (DAMPs), such as ATP and mitochondrial DNA (mtDNA) (46, 47). In the perception of PAMPs and/or DAMPs, NLRP3 recruits ASC and caspase-1 (48), which leads to caspase-1 activation, maturation, and secretion of pro-inflammatory cell molecules, such as IL-1β and IL-18, leading to pyroptosis (49, 50). NLRP3 inflammasome is widely considered to be ideal drug target for sepsis.

**Figure 5:**
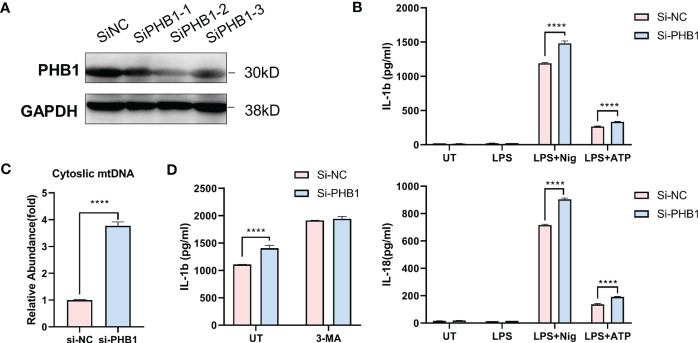
PHB1 inhibits the activation of NLRP3 inflammasome by regulating mitophagy. **(A)** Immunoblot analysis of extracts from mouse peritoneal macrophages silenced of PHB1. **(B)** ELISA of IL-1β (upper panel) and IL-18 (lower panel) in supernatants from mouse peritoneal macrophages silenced of PHB1, treated with indicated stimuli. **(C)** DNA isolated from cytosolic extracts was subjected to SYBR Green-based qPCR to quantitate mitochondrial DNA using specific primers. **(D)** ELISA of IL-1β in supernatants from mouse peritoneal macrophages silenced of PHB1, treated with 3-MA or/and Nigericin. Plots show the mean ± SD of technical replicates and are representative of at least three independent experiments; ****P<0.0001.

To verify the regulatory effect of core mitophagy molecule PHB1 on NLRP3 inflammasomes, we knocked down PHB1 in isolated macrophages (**Figure 5A**) and stimulated LPS priming cells with two classical agonists of NLRP3 inflammasomes, ATP and Nigericin. We found that the pro-inflammatory factors IL-1 β and IL-18 released by the cells were significantly increased after knocking down PHB1 (**Figure 5B**). At the same time, we also found that mitochondrial DNA (mtDNA) in the cytoplasm was increased after knocking down PHB1 (**Figure 5C**), suggesting that mitophagy might be inhibited. After we treated the cells with the autophagy inhibitor 3-MA, we found that the pro-inflammatory factors IL-1β released by the cells were not significantly different between the PHB1 knockdown group and the control group (**Figure 5D**). The above experimental results proved that PHB1 could inhibit the activation of NLRP3 inflammasomes by regulating mitophagy, which was highly consistent with our data analysis results.

### 3.6 Mining of small molecule drugs binding to PHB1

**Figure 6:**
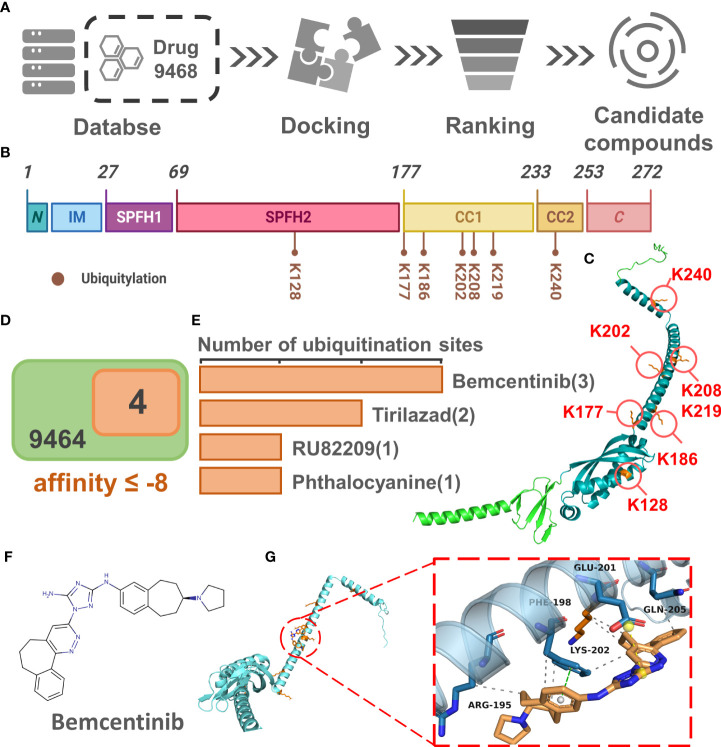
Identification of small molecular drugs targeting PHB1. **(A)** Small molecular drugs mining flow chart. **(B)** Domain organization of *H*. *sapiens* Prohibitin protein. **(C)** Venn diagram of drug screening. **(D)** Number of ubiquitination sites with binding affinity ≤ -7 for the four top drugs. **(E)** Protein conformation of PHB1 containing ubiquitination site, the blue structural sequence of protein is SPFH2, CC1 and CC2. **(F)** The chemical structure of Bemcentinib. **(G)** The binding model of PHB1 and Bemcentinib with the best affinity to ubiquitination site. The left panel shows the global view of PHB1 binding to ligands, and the right panel shows the focused view of binding sites.

**Figure 7:**
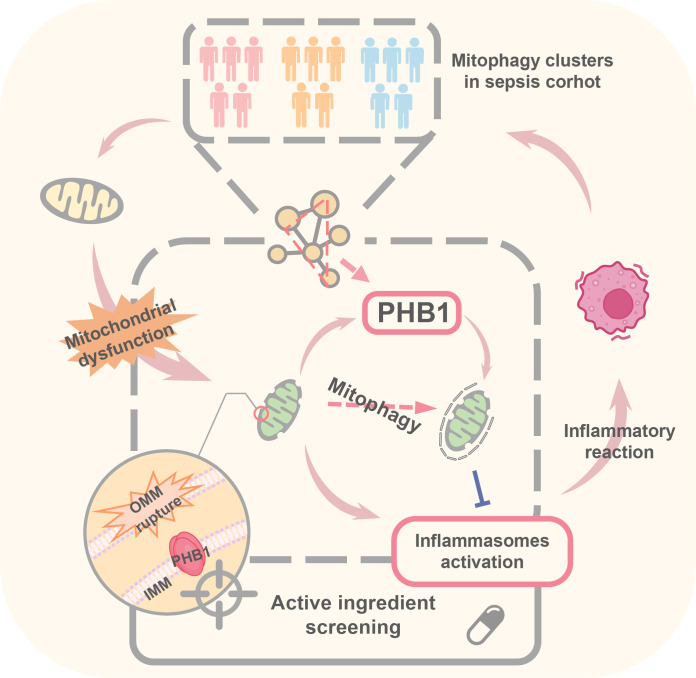
Pattern diagram of PHB1-mediated mitophagy in a septic cohort.

The results of the above analysis have shown that PHB1 plays an important role in sepsis. To further screen for small molecule drugs that might target PHB1 to exert anti-inflammatory activity, we tested the affinity of PHB1 with 9,468 drugs from DrugBank database (**Figure 6A**). Here, we first identified the protein domain of PHB1 through literature retrieval and structural prediction (**Figures 6B, C**). Since PHB1 does not belongs to a kinase protein (51), its activity cannot be directly activated by the substrate. Therefore, we attempted to screen out drugs that can increase the stability of its protein by binding to its ubiquitination site to further regulate mitophagy. Further analysis showed that the ubiquitination sites were mainly concentrated in the SPFH2, CC1, and CC2 sequences (**Figures 6B, C**), so we limited the docking region to these three sequences. After simulated docking, we found that only nine of the 9,468 drugs had an affinity of ≤-8 for the optimal conformation in which they combined with PHB1 (**Table S1**). Further screening showed that four drugs among the 9 drugs had conformations that combined with the ubiquitination site of PHB1 with affinity ≤-8, they are Bemcentinib, Tirilazad, RU82209 and Phthalocyanine (**Figures 6D, E**, **S1**-**5**). Among them, Bemcentinib had the most predicted binding sites (K202_-8.0 kcal/mol, K177_-7.7 kcal/mol, K128_-7.5 kcal/mol, K128_-7.2 kcal/mol) (**Figures 6F, G** and **S2**), while the other three drugs also had superior affinity for the ubiquitination sites, Tirilazad (K186_-8.0 kcal/mol, K128_-7.6 kcal/mol) (**Figure S3**). RU82209 (K177_-8.1 kcal/mol, K177_-7.0 kcal/mol) (**Figure S4**); Phthalocyanine (K186_-8.2 kcal/mol) (**Figure S5**). In addition, predictions have shown that Bemcentinib and Phthalocyanine are capable of hydrogen bond interactions with residues K128 and K186, respectively (**Figures S2**, **5**), while RU82209 is capable of π - Cation interaction with residue K177 (**Figure S4**). In conclusion, we have identified four small molecule drugs that may affect the ubiquitination modification of PHB1 protein to exert anti-inflammatory activity (**Figure 7**).

## 4 Discussion

Sepsis, with a high incidence and extremely complex pathogenesis, is closely related to the multi-system and multiple organ dysfunction syndrome, and has become a serious public health burden worldwide (10–12). A recent global analysis report on sepsis showed that in 2017, 11 million deaths were reported among 48.9 million cases recorded worldwide, accounting for 19.7% of all deaths worldwide (52). This means that sepsis is still one of the major health problems in the world. Massive release of inflammatory factors and enhanced oxidative stress response are important features of sepsis, and the alleviation of early excessive inflammatory response in sepsis can effectively control the progression of sepsis (53). However, due to the multi-source pathogenesis of sepsis and its complex host reaction, it is currently difficult to achieve standardized treatment for sepsis (54). Although a variety of drugs targeting inflammation-related sites have been developed, such as drugs targeting TNF-α, IL-1β or Toll-like receptors, satisfactory clinical effects have not been achieved (55, 56). The imbalance of body homeostasis and immunologic dysfunction have also prompted some current treatment directions to focus on the rescue of immunologic function (56). Thymosin alpha1, as an immunomodulator, can improve the prognosis of sepsis patients either alone or in combination with anti-inflammatory therapy (57). Considering the coexistence of extensive inflammatory response and immunosuppression, the combination therapy mode of anti-inflammation and immune enhancement can further improve the clinical effect (58, 59). In addition, since the balance and diversity of intestinal microorganisms are associated with the enhanced immunity of the body, the regulation of intestinal microorganisms also shows a certain therapeutic potential for sepsis (60). Nevertheless, the unique pathological characteristics of sepsis and multiple complications following a widespread inflammatory response have led to the limitation of single drug therapy (54), which urges researchers to still need to further subdivide the pathogenesis of different subsets to formulate individualized treatment. Therefore, it is of great significance to further explore the differences between different subsets of sepsis.

Mitophagy, as a selective autophagy, maintains mitochondrial homeostasis to prevent the accumulation of damaged mitochondria caused by excessive inflammatory reactions (1, 2). Many studies have reported that mitophagy can alleviate the excessive inflammatory response of inflammatory diseases and reduce cell death and organ damage (61, 62). In addition, as a sensor of the innate immune system, inflammasomes can trigger the induction of inflammatory response in the case of infection and cellular stress (63). In sepsis, the abnormal activation of inflammatory bodies leads to the activation of Caspase-1, which further matures and releases IL-1β and IL-18 (47, 64). PINK1, as a key regulator of ubiquitin-dependent mitophagy regulated by PINK1-Parkin pathway (16), can inhibit the activation of NLRP3 through mediated mitophagy, and thus reverse the cellular inflammatory damage (62). On this basis, we speculate that sepsis may be associated with mitophagy dysfunction to some extent.

To explore the role of mitochondrial autophagy in sepsis, we first investigated the expression of mitophagy-related pathways in different samples of sepsis and the immune correlation, and found that there were differences in mitophagy levels among different severity of sepsis pathological conditions. The mitophagy level in ICU patients was lower than that in ER patients. The mitophagy level in patients with higher SOFA score was generally lower, suggesting that a high mitophagy level may predict a good prognosis for sepsis patients After clustering the samples, we found that the samples could be stably divided into three clusters with different degrees of mitophagy, and the clinical indicators of the three subtypes were also different, which further suggested that the activation level of mitophagy reflected the clinical pathological state of sepsis to some extent. Next, we further examined the differences in the host immune characteristics at different levels of activation of mitophagy. We found that the enrichment levels of immune cells from different origins were different in different clusters, which further proved that maintaining mitochondrial function and activity is a prerequisite for the immune system homeostasis. While the dysfunction of mitophagy leads to the over-activation of inflammatory signaling pathways, and thus leads to the imbalance of immune function (65).

In order to find the key regulatory factors related to mitophagy, through modular clustering analysis on sepsis samples, we further analyzed the obtained 22 key genes related to mitophagy, and further analysis revealed that PHB1 gene is one of the core genes of the overall network. PHB1 belongs to the SPFH protein family and is a highly evolutionarily conservative protein that commonly exists in eukaryotic cells. PHB1 plays a key role in many aspects of mitochondrial biology (66), such as degradation of mitochondrial respiratory chain subunits, mitochondrial biogenesis, assembly and activity of oxidative phosphorylation systems, mitophagy, and mitochondrial apoptosis (67). The PHB protein complex found in mitochondria consists of two subunits, PHB1 and PHB2, which located in the mitochondrial inner membrane through physical interaction (68). They act on the mitophagy degradation process. The complex of PHB1 and PHB2 interacts with the autophagosome membrane-related protein LC3 to promote mitophagy (42). However, the related reports about the role of PHB1 in sepsis are still limited.

Our results showed that PHB1 was negatively correlated with the severity of sepsis patients, and the expression of PHB1 was also negatively correlated with the expression of NLRP3 inflammasomes. Abnormal activation of NLRP3 inflammasomes has been widely considered as ideal drug targets for sepsis because of their important roles in the pathogenesis and progression of sepsis (42, 47). The experiments *in vitro* proved that PHB1 gene could inhibit the activation of NLRP3 inflammasomes, depending on the mitophagy pathway. These results suggest that PHB1 has great potential as an intervention target for sepsis. The role and mechanism of PHB1 in sepsis will be further verified in gene knockout mice in our future work.

As PHB1 belongs to one of the members of SPFH protein family and is essential for mitochondrial kinetics and metabolic regulation, and the development of small molecule drugs targeting PHB1/PHB2 currently also shows a certain prospect for the treatment of metabolism and inflammatory diseases (69), we further studied the protein domain of PHB1 in order to find small molecule drugs that might regulate the activity of PHB1 protein. The PHB1 protein has a single N-terminal TM helix, and three core features of the SPFH protein, (two conserved SPFH domains (SPFH1,SPFH2), a Coiled-Coil domain (CC1,CC2)), and a C-terminal domain (51), where the PFH1 domain together with the N-terminal hydrophobic helix forms a membrane domain. Interactions with membrane lipids may hinder the binding of residues within the membrane domain to small molecule drugs (70). In addition, it can be found that all ubiquitination sites of PHB1 are exposed outside the membrane structure domain, and the integrity of the Coiled-Coil Region of PHB1 is also necessary to maintain the function of PHB1 (71), which also implies that small-molecule drugs may be more inclined to bind to motifs outside the membrane structure domain to exert the regulation of PHB1 expression.

We finally found four drugs (Bemcentinib, Tirilazad, RU82209, Phthalocyanine) that may bind to the ubiquitination site of PHB1 protein to increase the stability of the protein in the batch screened drugs. Among them, we learned that Bemcentinib has been currently studied for the treatment of non-small cell lung cancer, and the analysis results showed that Bemcentinib can bind to multiple ubiquitination sites (K202, K128, K177) of PHB1 with superior affinity (≤ -7). Interestingly, Bemcentinib is also an inhibitor of Spike glycoprotein, this drug was previously reported for the treatment of COVID-19 (72), which also suggests that Bemcentinib may be a small molecule drug with potential anti-inflammatory activity for the treatment of sepsis. Whether these drugs can effectively act on PHB1 needs further experimental verification. Nevertheless, our work still provides clues and certain reference values for clinical drug screening and subsequent drug development. In subsequent studies, we will further screen candidate compounds with anti-inflammatory activity targeting PHB1 through relevant experiments.

## Data availability statement

The original contributions presented in the study are included in the article/**Supplementary Material**. Further inquiries can be directed to the corresponding author.

## Author contributions

FL conceived the project and designed experiments and wrote the paper. SC analyzed the data and wrote the paper. JM and PY performed the experiments and assisted in data interpretation. All authors contributed to the article and approved the submitted version.
