# Supplementary material for: The landscape of mitophagy in sepsis reveals PHB1 as an NLRP3 inflammasome inhibitor
Source: Front Immunol. 2023 Jun 8;14:1188482. doi: 10.3389/fimmu.2023.1188482 (PMC10285102; doi:10.3389/fimmu.2023.1188482)
Supplement: Supplementary file 1 [file DataSheet_1.docx]

Supplementary Material

The landscape of mitophagy in sepsis reveals PHB1 as an NLRP3 inflammasome inhibitor

**Shipeng Chen^1,2†^, Jinqi Ma^3†^, Ping Yin^3^, Fang Liang^1^***

*** Correspondence:** Fang Liang. liangfang924@163.com

^†^ These authors contributed equally to this work and share first authorship

# **Supplementary Figures and Tables**

## Supplementary Figures


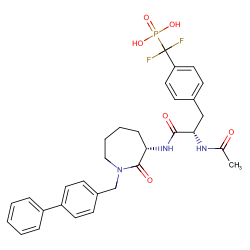

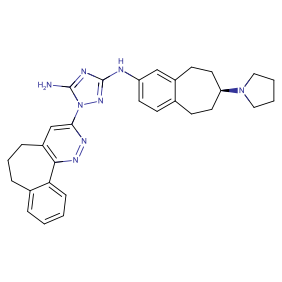


**Bemcentinib**


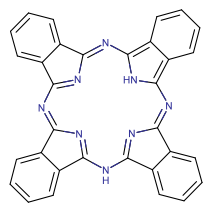


**Phthalocyanine**


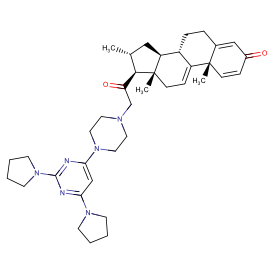


**Tirilazad**

**RU82209**

**(A)**

**(B)**

**(D)**

**(C)**

**Supplementary Figure 1.** Chemical structures of four candidate compounds. **(A)** Bemcentinib. **(B)** Tirilazad. **(C)** RU82209. **(D)** Phthalocyanine.


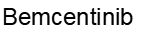

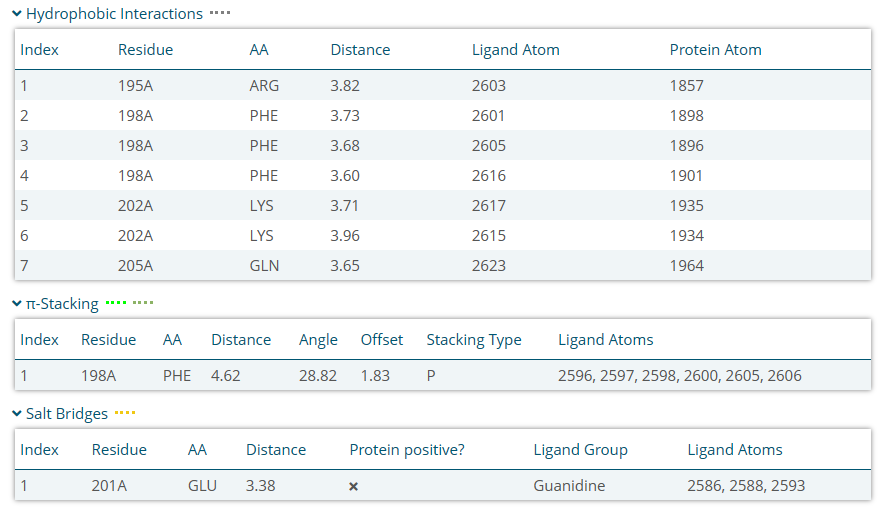

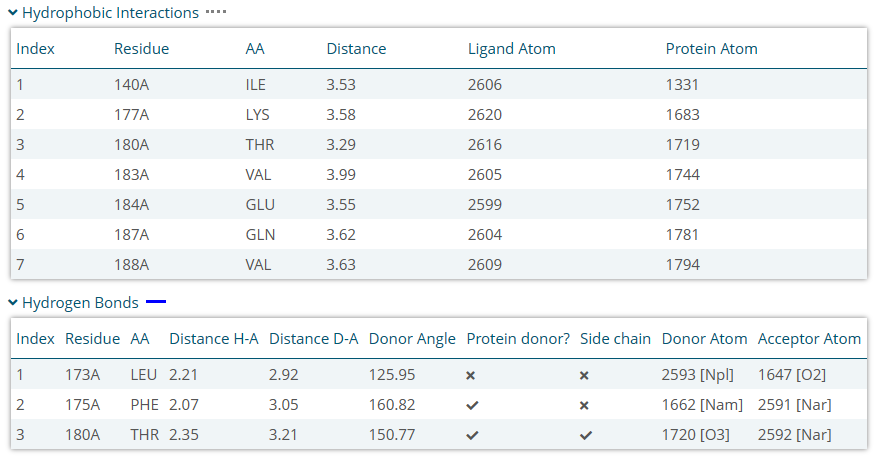

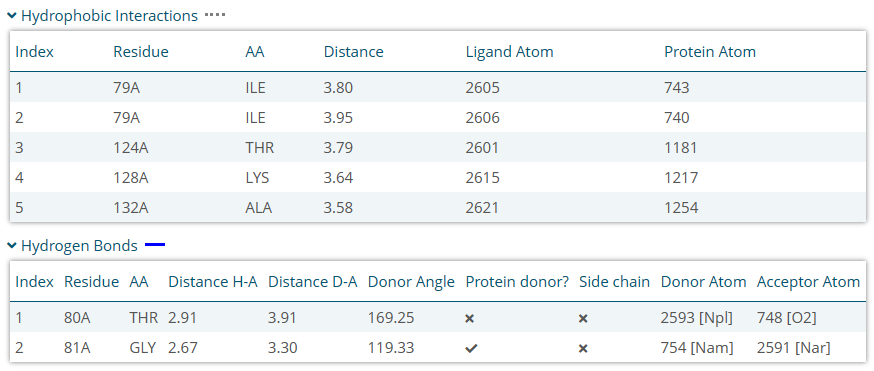

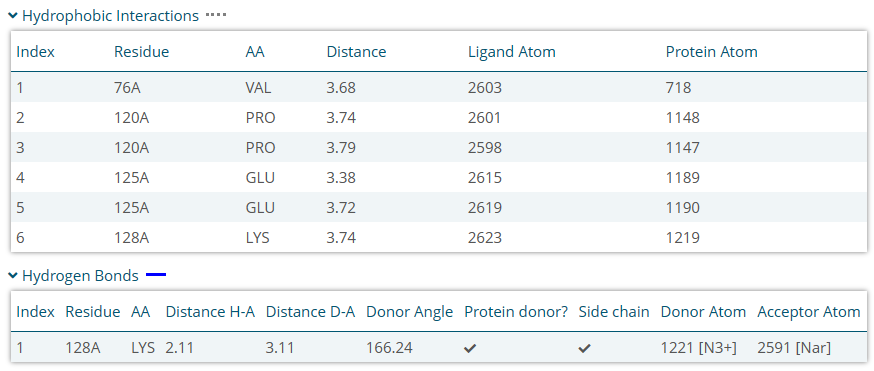


***K202***

**(affinity = -8.0 kcal/mol)**


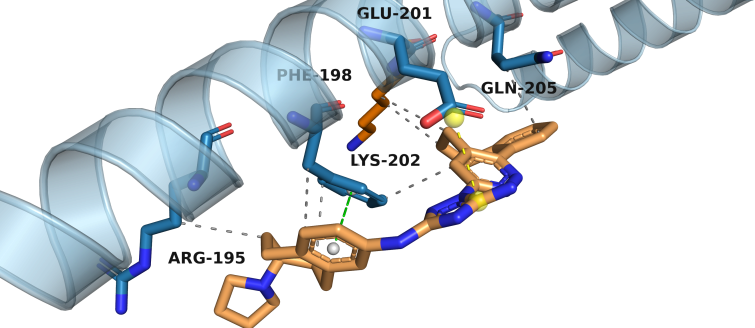

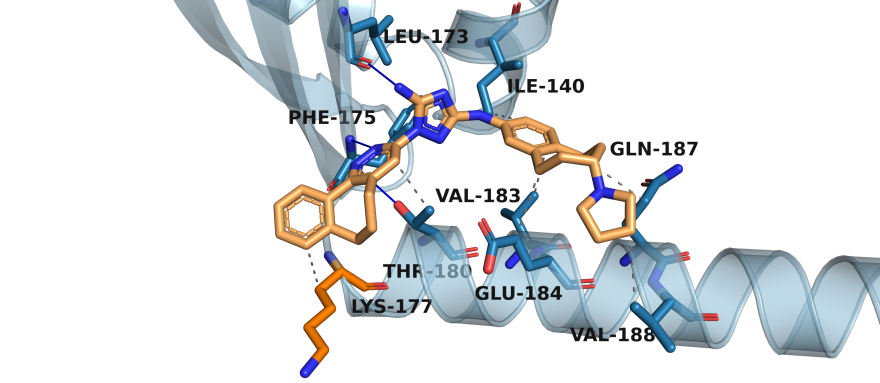


***K177***

**(affinity = -7.7 kcal/mol)**


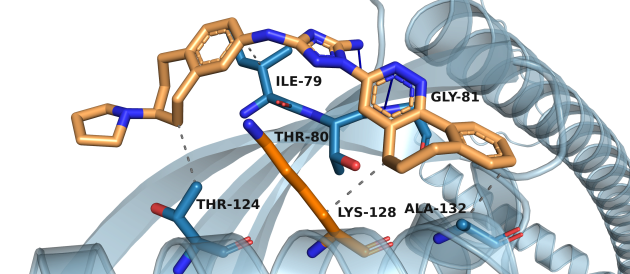


***K128***

**(affinity = -7.5 kcal/mol)**


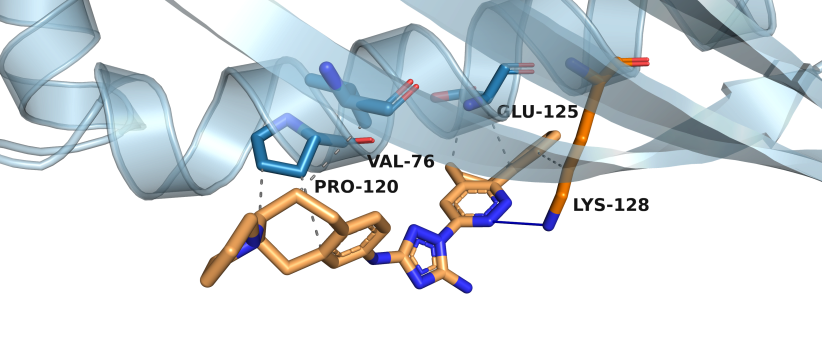


***K128***

***(affinity = -7.2 kcal/mol）***

**Supplementary Figure 2.** Conformation of Bemcentinib binding to different ubiquitination sites of PHB1. The left panel provides visualization of protein-ligand interactions and the right panel provides detailed information on residue-ligand interactions.


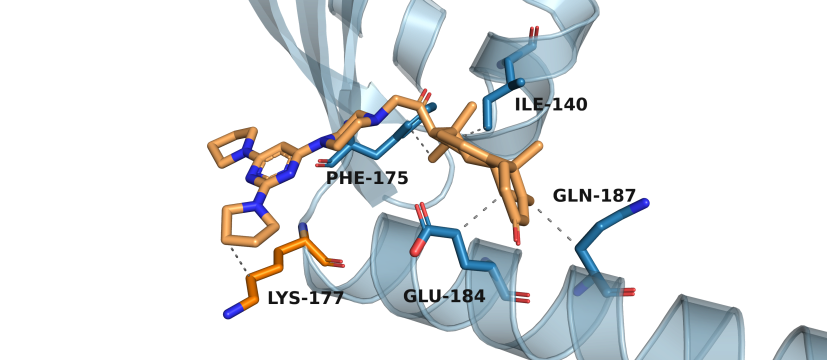

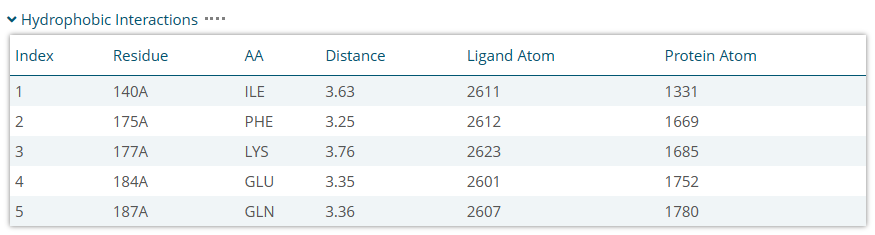


**K186**

**(affinity = -8.0 kcal/mol)**


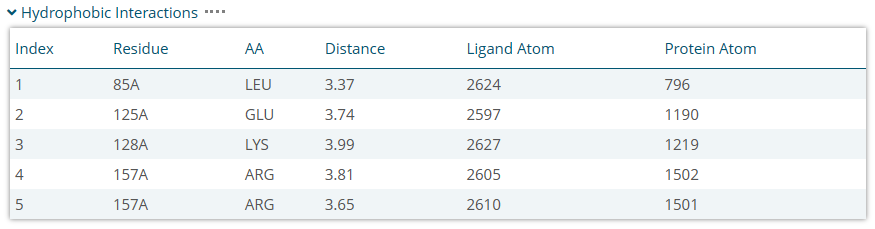


**K128**

**(affinity = -7.6 kcal/mol)**


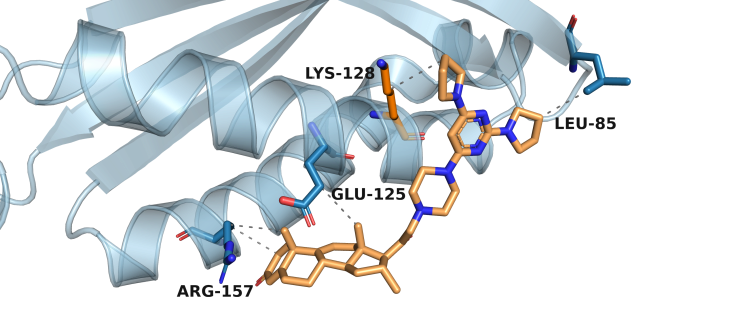


Tirilazad

**Supplementary Figure 3.** Conformation of Tirilazad binding to different ubiquitination sites of PHB1. The left panel provides visualization of protein-ligand interactions and the right panel provides detailed information on residue-ligand interactions.

**K177**

**(affinity = -8.1 kcal/mol）**


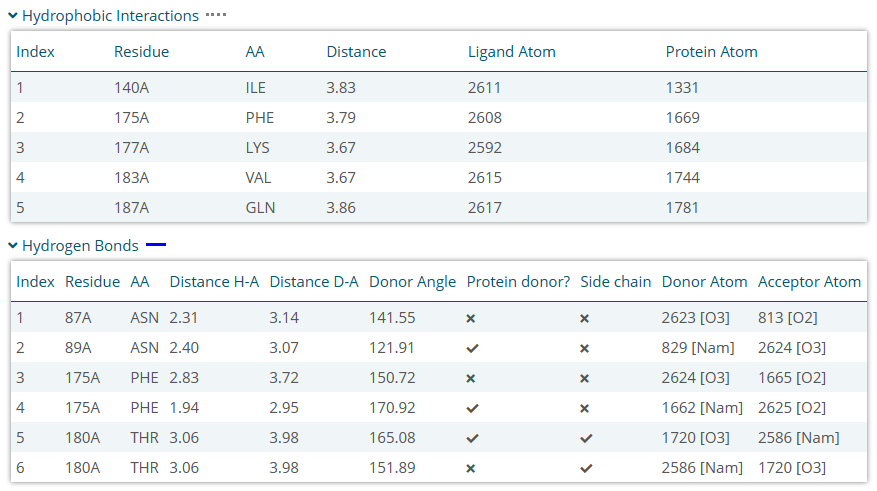

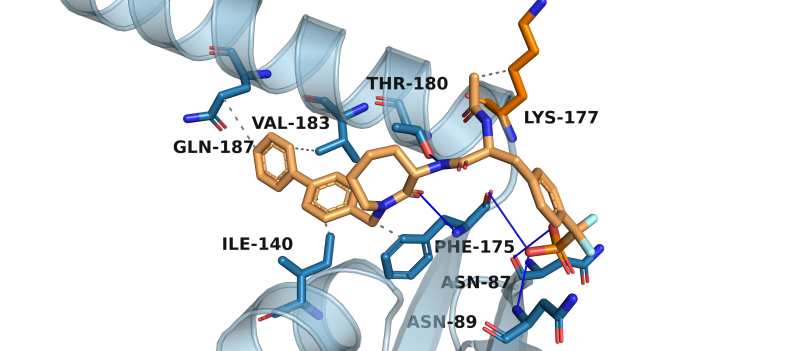

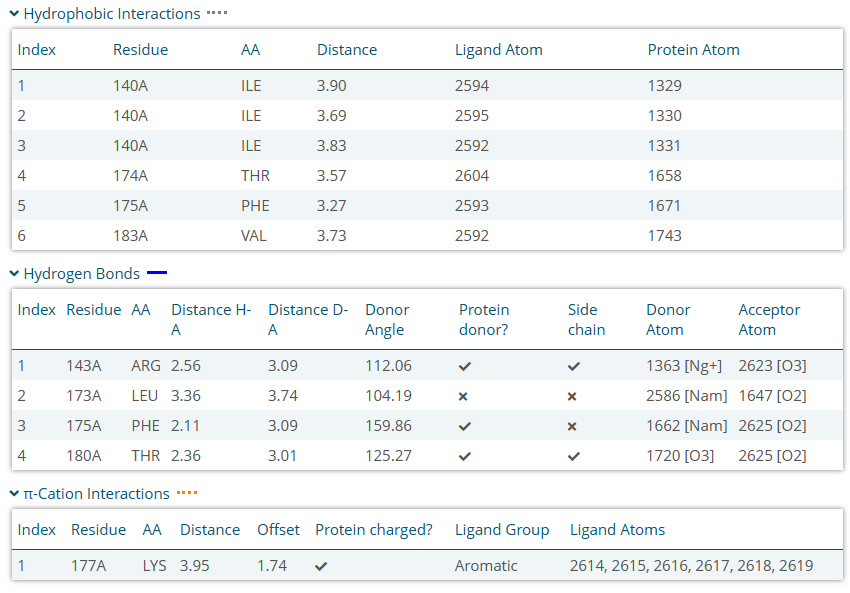


**K177**

**(affinity = -7.0 kcal/mol)**


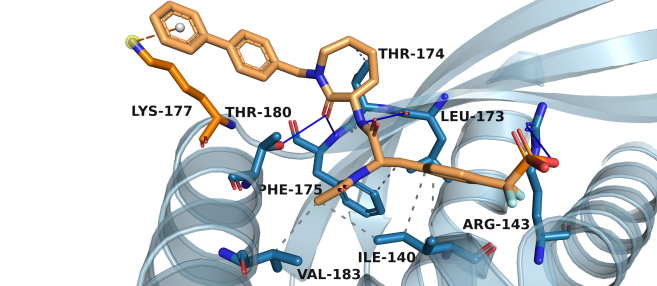


RU82209

**Supplementary Figure 4.** Conformation of RU82209 binding to different ubiquitination sites of PHB1. The left panel provides visualization of protein-ligand interactions and the right panel provides detailed information on residue-ligand interactions.


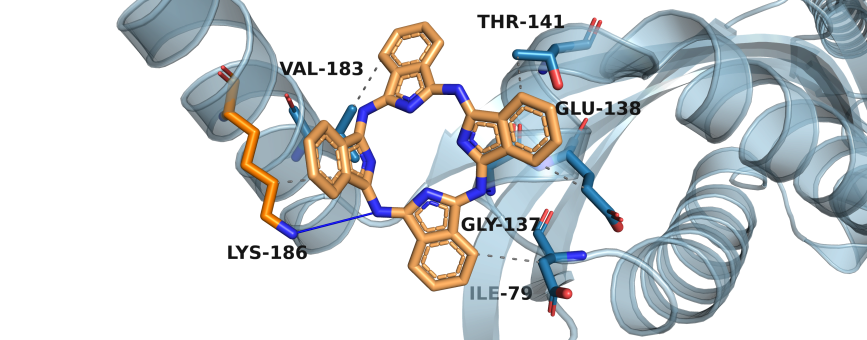

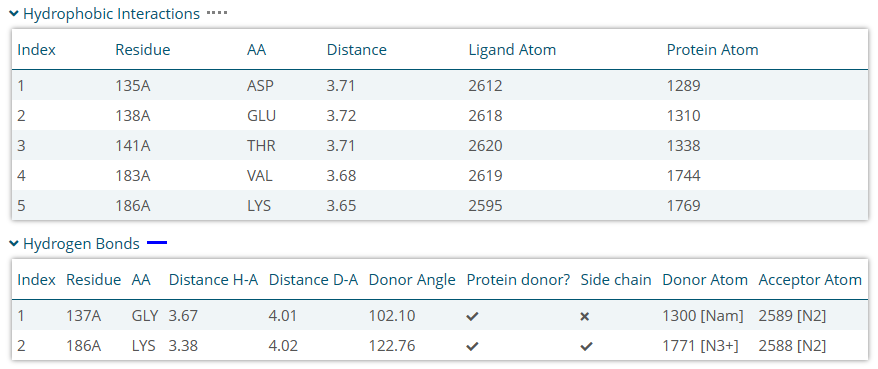


**K186**

**(affinity = -8.2 kcal/mol)**

Phthalocyanine

**Supplementary Figure 5.** Conformation of Phthalocyanine binding to different ubiquitination sites of PHB1. The left panel provides visualization of protein-ligand interactions and the right panel provides detailed information on residue-ligand interactions.

## Supplementary Tables

| **ID** | **Optimal affinity** | **DRUG_GROUPS** | **GENERIC_NAME** |
| --- | --- | --- | --- |
| Drug_3750 | -8.5 kcal/mol | approved; investigational | Nilotinib |
| Drug_7125 | -8.3 kcal/mol | investigational | Radotinib |
| Drug_7558 | -8.2 kcal/mol | investigational | Phthalocyanine |
| Drug_7599 | -8.1 kcal/mol | investigational | Tirilazad |
| Drug_2752 | -8.1 kcal/mol | experimental | RU82209 |
| Drug_7185 | -8.1 kcal/mol | investigational | Bemcentinib |
| Drug_3989 | -8.1 kcal/mol | investigational | RAF-265 |
| Drug_3084 | -8.0 kcal/mol | experimental | DB04016 |
| Drug_7195 | -8.0 kcal/mol | investigational | MK-3207 |

**Supplementary Table 1.** Nine drugs with optimal affinity ≤ -8 for binding to PHB1.
